# Optical diffractometry by rough phase steps

**DOI:** 10.1038/s41598-023-40267-6

**Published:** 2023-08-12

**Authors:** Morteza Jafari Siavashani, Elyas Nasimdoust, Parviz Elahi, Mohammad Taghi Tavassoly, Ali-Reza Moradi

**Affiliations:** 1 Department of Physics, Sharif University of Technology, Tehran, 11155-9161 Iran; 2 Department of Physics, Institute for Advanced Studies in Basic Sciences (IASBS), Zanjan, 45137-66731 Iran; 3 Department of Physics, Bogazici University, Bebek, 34342 Istanbul, Turkey; 4 Department of Physics, College of Science, University of Tehran, Kargar Shomali Ave, Tehran, 14399-55961 Iran; 5 School of Nano Science, Institute for Research in Fundamental Sciences (IPM), PO Box 19395-5531, Tehran, 19395 Iran

**Keywords:** Optical metrology, Optical physics

## Abstract

Optical diffractometry (OD) using a phase step is an alternative for interferometry, further, has least sensitivity to environmental vibrations. Therefore, OD has found numerous interesting metrological and technological applications. OD utilizes a phase step to detect the influence of objects under measurement by the changes in the Fresnel diffraction pattern. Recently, we showed that such measurements do not require infinitively sharp phase steps, although fabrication of such sharp elements is also impossible. Here, we address the issue of smoothness of the phase step surfaces. So far, in all of the OD applications the surfaces of the incorporated phase steps are considered to be optically smooth and flat. However, practically, some amount of roughness and unflatness is unavoidable even in precise and careful fabrication process. We show that preserving the OD-diffraction-pattern characteristics of a phase step depends on the level of roughness in the surfaces of the phase step. We define number of detectable fringes and autocorrelation functions of the diffraction patterns as the measures for evaluating the similarity of the rough phase step diffractions to the ideal case. We derive the theoretical description and confirm the results with simulations and experiments.

## Introduction

An abrupt change or confinement in the phase, amplitude, phase gradient, or polarization state of a light wavefront causes appreciable Fresnel diffraction, and the diffraction pattern includes information of the diffracting object^1–5^. The technique of “Optical Diffractometry (OD)” extracts such information, which can be on the object’s light absorption behavior, optical phase changes, or polarization characteristics. OD can be applied either in reflection from a reflective physical step, or in transmission by passing light through a boundary region of transparent media with different refractive indices. Mostly OD is used with visible light, but it can be also performed by other wave sources such as X-ray^6^. Using wave optics analysis OD is formulated and studied rather comprehensively in both reflection and transmission modes^2,3^. However, recently M. T. Tavassoly showed that Fresnel diffraction is a basic quantum mechanical effect^7^. In another interpretation, the phase step diffraction fringes may be considered as a hologram of the interfering light waves leaving the two sides of the phase step^8^.

The visibility of the diffraction fringes and the positions of its extrema usually serve as criteria in the aforementioned measurements^2,9^. These parameters vary as the optical path difference (OPD) changes, which in turn is resulted from variations in the incidence angle of light, height of the phase step, refractive index of the object, or refractive index of the surrounding medium (in transmission mode)^2^.

Considering the robustness again vibrations, feasibility, and other advantages over optical interferometry, OD from phase steps has found several absorbing metrological and technological applications. Amongst, precise measurement of displacements at the scales down to nanometer^10^, thin-film thickness^11^, refractive indices of solids and liquids^12,13^, diffusion coefficient^3^, temperature gradient^14^, etching rate^15^, coherence parameters and spectral line-shape^16^, direct measurement of the x-ray refractive index^6^, color dispersion^17^, wavemetry^18^, and quantitative 3D phase imaging^9^ have been the most effective ones to name.

In the predated OD applications as well as in theoretical studies, the phase step always was considered to be a sharp step. Whilst it is certainly impossible to fabricate such sharp step and a level of bluntness is unavoidable. Recently, we investigated the impact of bluntness of the phase steps on the OD measurements^1^. We specifically proved that up to 10% bluntness can be tolerated in phase-step based OD without considerable effect on the measurements. The bluntness parameter may be defined as the ratio of the conjunction length of the phase step to its height.

Here, we address another important issue, which is the smoothness of the phase step surfaces. Likewise to the case of sharpness, practically fabrication of a 100% optically flat and smooth phase step is impossible. The residual roughness, in turn, cause an overlaid random field, called speckle pattern, on the Fresnel diffraction. It is remarkable that in several imaging and detection methods usually the presence of speckle is considered as a source of error and a disturbing factor. However, proper statistical processing of the intensity distribution of the speckle field can provide valuable overall information about the dynamic changes on the scatterer object from which the speckle field is originated^19–25^. Nevertheless, here we focus on the aforementioned disturbing feature of the speckle phenomenon.

## Theory and simulations

**Figure 1:**
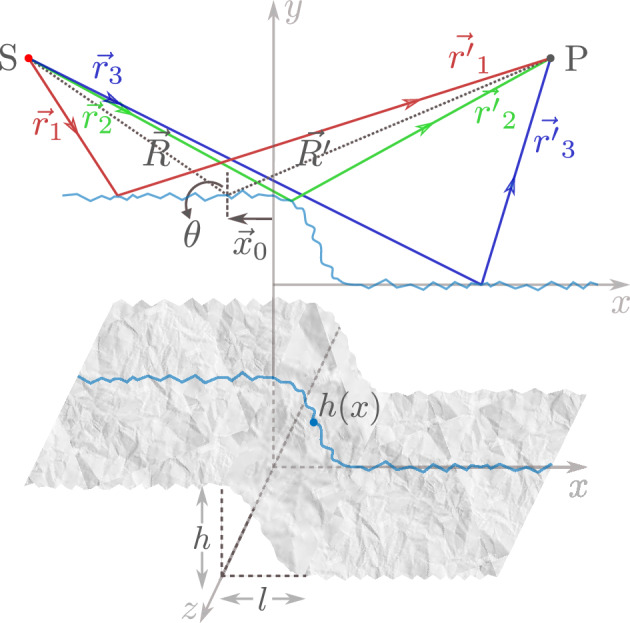
Scheme of Fresnel diffraction formation from a rough phase step.

In order to simplify the theoretical investigation the one-dimensional (1D) phase step is considered. As schematically shown in Fig. 1, incidence of a cylindrical wave-front originating from a linear source S on the 1D phase step, which has the symmetry axis perpendicular to the page and passes through S, forms the Fresnel diffraction. P is an arbitrary observation point. The difference between the height of the two parallel surfaces is *h* and the length of the conjunction is *l*. In Fig. 1 the origin of the coordinate system is defined on the upper side of the phase step where the conjunction starts. Therefore, $y(x=0)$ equals to *h* and $y(x=l)$ equals to zero. Using the Fresnel-Kirchhoff integral the diffracted complex amplitude and intensity can be calculated^26^. The integration is performed over all the rays originating from the secondary Huygens sources formed upon striking rays on the phase step. Three representing rays originating from the source point S and incident on left ($\vec{r}_1$), conjunction ($\vec{r}_2$), and right ($\vec{r}_3$) parts of the step, reach to the point P by $\vec{r^{\prime}}_1$, $\vec{r^{\prime}}_2$, and $\vec{r^{\prime}}_3$, respectively. The incident point of the specular reflection is shown by the vector $\vec{x}_0$, and $\vec{R}$ and $\vec{R^{\prime}}$ are the incident and reflected rays to this point, respectively. The complex amplitude of the diffracted wave-front in point P is calculated by taking the diffraction integral throughout the phase step, which leads to:

1 $$\begin{aligned} u(\textrm{P}) = \sqrt{\frac{-i}{\lambda }} A {\mathfrak {R}} \frac{e^{{i k (R+R^\prime )}}}{{\sqrt{R R^\prime }}} \int _{-\infty }^{+\infty } \textrm{d}x \, e^{i k \frac{(x-x_0)^2}{2}(\frac{1}{R}+\frac{1}{R^\prime })+2 i k h(x) \cos {\theta }}, \end{aligned}$$

where $\lambda$, $\sqrt{\frac{-i}{\lambda }}$, *A*, $k=2\pi /\lambda$ and ${\mathfrak {R}}$ are the wavelength, inclination factor, amplitude of incident wave, wave number, and the amplitude reflectance of the phase step surface, respectively. In obtaining the above integral the Fresnel approximation is considered, and the vectors $\vec{r}_1$, $\vec{r}_2$, $\vec{r}_3$, $\vec{r^{\prime}}_1$, $\vec{r^{\prime}}_2$, and $\vec{r^{\prime}}_3$ were expressed in terms of $\vec{R}$, $\vec{R^{\prime}}$, $\vec{x}_0$, *h*(*x*), and $\theta$, which are depicted in Fig. 1. The details on derivation of Eq. (1) can be found in^1,2^.

**Figure 2:**
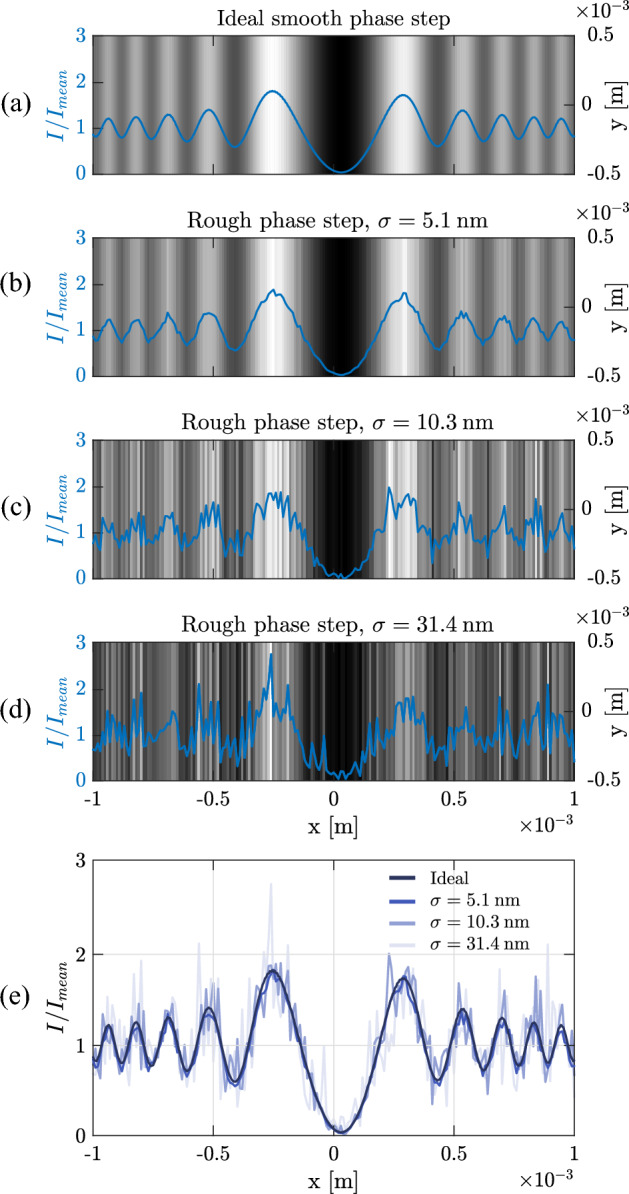
Simulation results of Fresnel diffraction from phase steps and the overlaid intensity profiles along the line perpendicular to the phase step direction for of different superimposed roughness values: (**a**) $\sigma =0$ (ideally smooth phase step), (**b**) $\sigma =5.1$ nm, (**c**) $\sigma =10.3$ nm, and (**d**) $\sigma =31.4$ nm. In these simulation $a=1$, $b=1$, and $c=4\times 10^8$. (**e**) The intensity profiles of the diffracted patterns along *x* axis for simulated phase steps of various roughness.

The function *h*(*x*) describes the geometry of the phase step. In the case of infinitively sharp and smooth surface phase step, indeed, this function is a step function. In a blunt and smooth phase step, as we discussed in^1^, this function may be approximated as a polynomial expansion of at least ${\mathcal {O}}$(5) (to fulfill the continuity and differentiability conditions). Alternatively, and in a more general view, Sigmoid-like interpolation and clamping functions, such as “Smoothstep” function can be also considered^27^. In the present study, we further consider that the surfaces of the phase step has a level of roughness as well. The roughness is also included in the geometry function *h*(*x*). However, in order to discriminate the effect of roughness of the surfaces from the effect of bluntness on the Fresnel diffraction from the phase steps, in our simulations and experimental investigations only a slight bluntness is considered; the bluntness in simulations is taken as $\frac{l}{h}=0.0015$, which according to^1^ applies negligible effect on the diffraction pattern. Additionally, it is considered that *h*(*x*) fulfills the continuity and differentiability conditions, i.e. its first and second derivatives is zero in the junctions. Similarly, in the associated experiments the phase steps has negligible bluntness.

Analytical calculation of Eq. (1) is not an easy task, therefore, numerical simulations have to be performed alternatively. The most straightforward consideration for the roughness is the additional noise function added to *h*(*x*):

2 $$\begin{aligned} h(x)=h~\mathrm{{rSig}}(x)+f_n(x), \end{aligned}$$

where $\mathrm{{rSig}}(x)$ is a reverse Sigmoid function to represent a smooth phase step:

3 $$\begin{aligned} \mathrm{{rSig}}(x)= \frac{a}{b+\exp {(-cx)}}, \end{aligned}$$

where *a*, *b*, and *c* are free adjusting parameters^27^. For a large value of *c*, $\mathrm{{rSig}}(x)$ approaches to a step function. In the simulation we take $a=1$, $b=1$, and $c=4\times 10^8$, which ensures neglecting the effect of edge bluntness on the diffraction pattern^1^. $f_n(x)=h_r~\mathrm{{Sprand}}(x)$, where $\mathrm{{Sprand}}(x)$ generates sparse random noises between 0 to 1 along the *x* direction^28^. The roughness sparsity is tuned by the density argument of this function and the parameter $h_r$ is multiplied to it as the strength parameter. Figure 2 shows the simulation results of Fresnel diffraction from phase steps with different roughnesses. In these simulations the step height is $h=20~\upmu$m and $\theta =0$. The distances *R* and $R^\prime$ are taken 50 cm in the simulations and in the experiments. From the *h*(*x*) function and based on the generated noise distributions the roughness of the phase steps surfaces are calculated as the standard deviation of the height distribution ($\sigma$). Figure 2a–d show the diffraction patterns and the overlaid intensity profiles along the line perpendicular to the phase step direction for $\sigma =$0, 5.1 nm, 10.3 nm, and 31.4 nm, respectively. It is observable that decreasing the smoothness (higher $\sigma$) affects the diffraction. The results qualitatively show that for $\sigma =31.4$ nm it is barely possible to attribute the pattern to a phase step diffraction pattern. It is remarkable that the simulation and analytical calculations are performed in 1D even if the 2D diffraction patterns are shown in Fig. 2. Therefore, the fringe-like structures in rough surface phase steps are indeed the extending of the applied roughness on the 1D profiles of the diffraction patterns. For the experimental results we will define measures to quantitatively express the similarity of the patterns to phase-step diffraction patterns, and assess the effect of surface roughness on OD. Therefore, the criteria can imply that whether the measurements based on the diffraction pattern analysis of a phase-step of known roughness is valid. In the Supplementary video S2 changing the cross-sectional profile of the diffraction patterns by varying $\sigma$ is shown. The procedure has applied on a phase step corresponding to an optical path difference of $\frac{\lambda }{4}$. This phase step has a symmetric diffraction pattern and the maximum visibility which leads to the most sensitivity to the applied roughnesses. This level of sensitivity, indeed, provides an additional feature of OD, to be used as a smoothness assessment tool or for externally induced noise detection and measurement.

**Figure 3:**
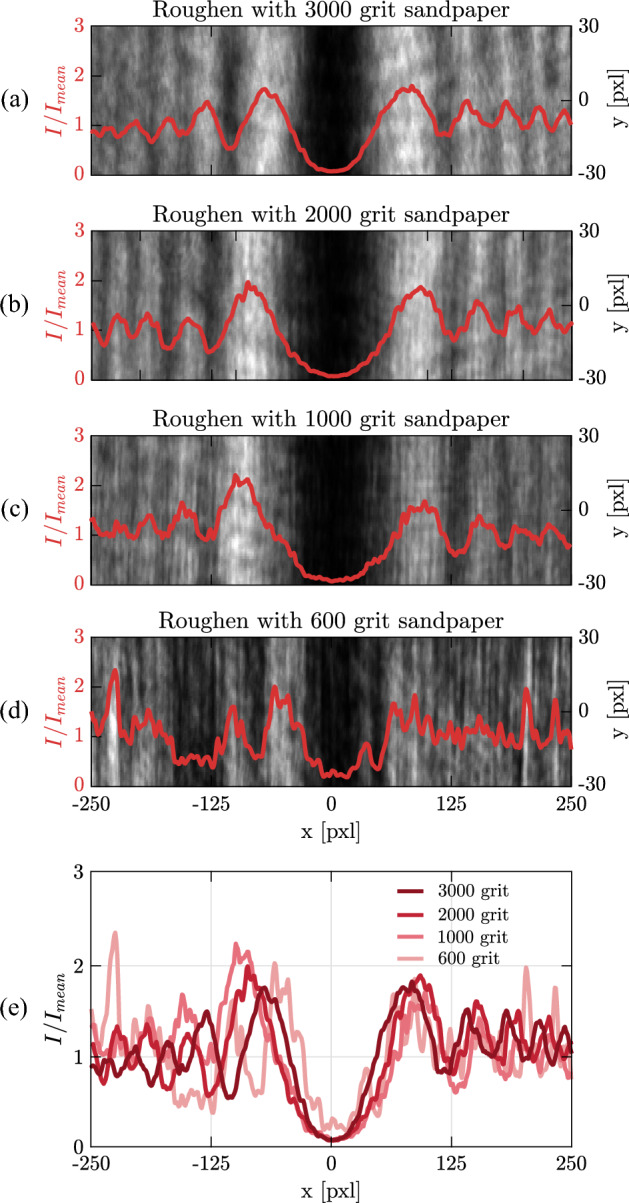
Fresnel diffraction and overlaid cross-sectional profiles of rough phase steps roughened by sanding smooth mirrors for 4 min by sandpapers with grit numbers of (**a**) 3000, (**b**) 2000, (**c**) 1000 and (**d**) 600. (**e**) The cross-sectional intensity profiles of the experimental diffracted patterns along *x* axis for phase steps of various roughness.

## Results and discussion

Figure 3a–d show the Fresnel diffraction from phase steps with four different roughnesses obtained by roughening the smooth mirrors for 4 min by the sandpapers of grit numbers 3000, 2000,1000 and 600, respectively. In Supplementary Fig. S1 similar patterns and overlaid cross-sectional profiles for the rest of grit numbers and for sanding times of 4 min and 6 min are shown. The results show that the shape of fringes can not be recognized for the phase steps roughened with sandpapers of grit number 600 and below. Within the available rough phase steps, this defines a threshold after which the phase step OD based measurements are not reliable anymore.

**Figure 4:**
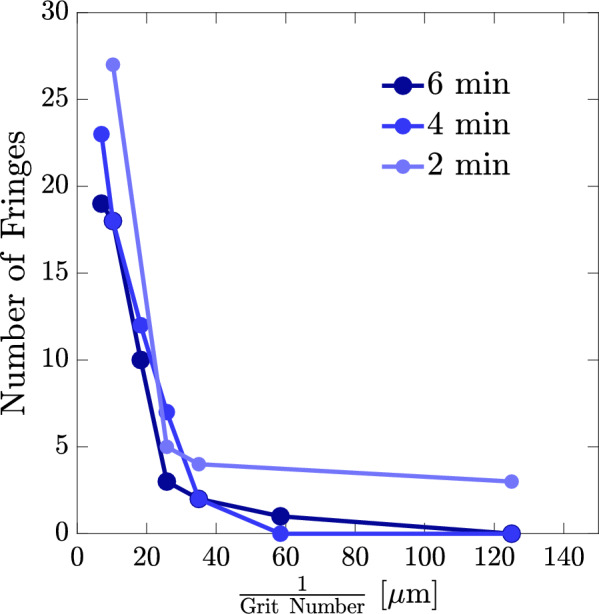
Number of fringes versus surface roughness ($\propto$ $\frac{1}{\mathrm{{Grit ~Number}}}$) for the roughened mirrors.

To assess the threshold for the OD measurements in a more quantitative fashion we extract two parameters from the recorded diffraction patterns: (1) number of recognizable fringes and (2) autocorrelation function. A typical diffraction pattern of a phase step includes a minimum surrounded by two maxima. Then, at both left and right sides fringes of smaller width and less maximum intensities appear. The number of fringes, or equivalently number of extrema of the recorded patterns versus the surface roughness ($\propto$ $\frac{1}{\mathrm{{Grit ~Number}}}$) for the roughened mirrors are shown in Fig. 4. In order to avoid the noises effect, counting the fringes are performed after smoothing the data. As expected, increasing the sanding time roughens the phase steps more and the number of recognizable fringes reduces. Also, for a constant sanding time smaller grit numbers results in higher surface roughness and, therefore, smaller number of recognizable fringes. For the examined sets of mirrors number of fringe counting shows that there are specific cases of different grit numbers and sanding times which results in equivalent statuses. For example, using sandpaper of grit number 3000 for 6 min equals to the case of grit number 2000 for 4 min rubbing, or using sandpaper of grit number 120 for 2 min is similar to the case of grit number 600 for 6 min rubbing.

**Figure 5:**
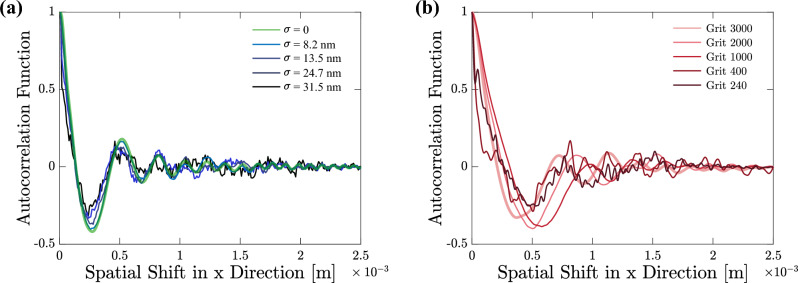
Autocorrelation function (ACF) of the OD cross-sectional profiles to evaluate the similarity of the roughened phase steps diffraction pattern to the ideal case. Calculated AFC for (**a**) the simulation diffraction patterns and (**b**) the experimental results.

Moreover, autocorrelation function (ACF) of the OD cross-sectional profiles may exhibit quantitatively the similarity of the roughened pattern to the ideal case more effectively. Figure  5a shows the calculated ACF for the simulation results. ACF is calculated as a function of spatial shift of the cross-sectional profiles of the diffraction patterns along the x-direction. The calculation is shown for the phase steps of $\sigma =0$ (ideally smooth phase step), $\sigma =5.1$ nm, $\sigma =10.3$ nm, and $\sigma =31.4$ nm. Similarly, Fig. 5b shows the ACF for the experimental cross-sectional profiles. As the spatial shift increases the similarity of the fringe patterns to the initial pattern reduces. Therefore, ACF damps into zero in the shifts beyond $\approx$2 mm for the smooth phase step (green color in Fig. 5a). As the roughness increases damping happens in a smaller spatial shift. This is even more pronounced in the experimental results, since the diffraction pattern contains more intensity distribution randomness and is not as symmetric as the theoretical case along the y-axis. The ACF results suggest a threshold beyond which the diffraction pattern is not similar to an OD pattern anymore. Accordingly, such rough phase steps cannot be utilized for OD based measurements. Nevertheless, there is no specific threshold to govern the experiments and this ACF criteria is a proximate guiding threshold to secure the measurements in the metrological or other applications of OD.

## Conclusion

In conclusion, we investigated the effect of surface roughness on phase-step OD. Since in all of the theoretical investigations and experimental measurements the utilized phase steps are considered to be ideally smooth, addressing the issue of phase step surface smoothness was a required task. We showed that diffraction pattern of a phase step will preserve its OD-diffraction-pattern characteristics if the level of roughness in the surfaces of the phase step does not exceed a definable threshold. The threshold can be defined by finding the similarity of the diffraction pattern to the smooth phase step case via quantitative parameters such as number of central fringes and ACF. We derived the theoretical explanations and presented simulation results. We finally performed experiments on rough phase steps by the use of a Michelson’s interferometry based variable phase step arrangement. The theoretical predictions and the experimental results are consistent. We expect that utilizing phase steps, of which one surfaces is a smooth mirror, has also the potential to be exploited, and it is the subject of an in-progress research. Also, considering other types of unflatness, such as waviness of the surfaces can be investigated. However, roughness of the surfaces seems to be the most common and effective incompleteness of surfaces.

## Methods

### Experimental procedure

By the use of lithography techniques or thin film deposition, phase steps of almost any desired height and excellent quality can be fabricated. However, on the one hand, such approaches are expensive and require high efforts, and on the other hand, fabrication of phase steps with chosen levels of roughness that we use in our experiments is a challenge. Moreover, despite simulations fabricating a phase step of adjustable height is an impossible task in usual fabrication techniques. To overcome the aforementioned shortcomings, based on the use of a modified Michelson’s interferometer, we provide an adjustable phase step system, which not only provides 1D phase step of any required height but also it yields 2D phase steps of various shapes. Moreover, applying roughness, bluntness, waviness or other imperfections on the phase steps is possible with this arrangement.

**Figure 6:**
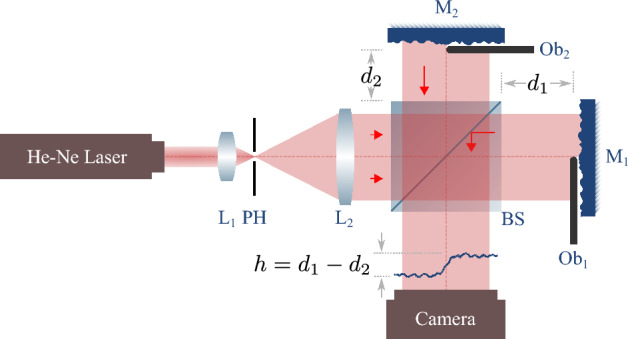
Schematic setup for variable step height OD experiments; *L* lens, *PH* pinhole, *BS* beam splitter, *M* mirror, *Ob* obstacle.

The scheme of the setup is presented in Fig. 6. It is a Michelson’s interferometer, illuminated by a spatially filtered and collimated He-Ne laser beam (632.8 nm), in which half of each mirror (M$_1$ and M$_2$) is covered by a sharp edge obstacle (Ob$_1$ and Ob$_2$). Therefore, in the output of the apparatus the beam splitter (BS) gathers the light of the two arms without any overlapping of the lights. Instead, the differences between the optical path lengths of the two arms ($d_1$ and $d_2$) in the output of the apparatus can be considered as a phase step with the height $h=d_1-d_2$. Therefore, by adjusting the arms difference it is possible to attain a variable height phase step. The adjustment can be performed with a translation resolution that is defined by the positioner of one of the mirrors. With the current available translation solutions sub-nanometer precisions on the step adjustment can be achieved. In order to avoid the effect of phase step bluntness, the obstacles include a couple of razor blades. A razor blade is considered as the sharpest obstacle with the conjunction length of about 1 $\upmu$m^29^. The mirrors’ positions are remained untouched to ensure of having a constant *h* throughout the experiments. A rough phase step is obtained by utilizing a pair of mirrors with a predefined roughness for the two arms. By the use of sandpapers with grit numbers of 3000, 2000, 1000, 600, 400, 240, and 120 and sanding in one direction for three different sanding times of 2 min, 4 min, and 6 min, twenty one pairs of roughen mirrors are obtained and used. The sanding is performed in a constant pressure and the sanding speed, i.e., the number of times the sandpaper is passed across the mirror when sanding is kept constant during the preparation process. The information about the twenty one phase steps are summarized in the Supplementary Table S1.

### Supplementary Information

Supplementary Information 1.

Supplementary Information 2.

## Data Availability

The datasets used and/or analysed during the current study available from the corresponding author on reasonable request.
