# Supplementary material for: Optical diffractometry by rough phase steps
Source: Sci Rep. 2023 Aug 12;13:13155. doi: 10.1038/s41598-023-40267-6 (PMC10423200; doi:10.1038/s41598-023-40267-6)
Supplement: Supplementary file 1 — Supplementary Information 1. [file 41598_2023_40267_MOESM1_ESM.pdf]

Supplementary Information

Optical diffractometry by rough phase steps

Morteza Jafari Siavashani[1], Elyas Nasimdoust[2], Parviz Elahi[3], Mohammad Taghi Tavassoly[4], Ali-Reza Moradi[1,5,*]

[1] Department of Physics, Sharif University of Technology, Tehran 11155-9161, Iran

[2] Department of Physics, Institute for Advanced Studies in Basic Sciences (IASBS), Zanjan 45137-66731, Iran

[3] Department of Physics, Bogazici University, Bebek 34342, Istanbul, Turkey

[4] Department of Physics, College of Science, University of Tehran, Tehran 14399-55961, Iran

[5] School of Nano Science, Institute for Research in Fundamental Sciences (IPM), Tehran 19395-5531, Iran

*Corresponding author: *moradika@iasbs.ac.ir*

Contents:

Supplementary Video V1

Video of the simulated diffraction patterns and their associated cross-sectional profiles vs. phase step roughness.

Supplementary Table T1

Information of the phase steps used in the experiments.

Supplementary Figure S1

Diffraction patterns and overlaid cross-sectional profiles of the experiments on different phase steps.

Supplementary Table T 1: Information of the phase steps; Mirrors roughened with sandpapers of different grit numbers and for different sanding times.

number	grit number	grain size	sanding time
1	3000	7 μm	2 min
2	3000	7 μm	4 min
3	3000	7 μm	6 min
4	2000	10.3 μm	2 min
5	2000	10.3 μm	4 min
6	2000	10.3 μm	6 min
7	1000	18.3 μm	2 min
8	1000	18.3 μm	4 min
9	1000	18.3 μm	6 min
10	600	25.8 μm	2 min
11	600	25.8 μm	4 min
12	600	25.8 μm	6 min
13	400	35 μm	2 min
14	400	35 μm	4 min
15	400	35 μm	6 min
16	240	58.5 μm	2 min
17	240	58.5 μm	4 min
18	240	58.5 μm	6 min
19	120	125 μm	2 min
20	120	125 μm	4 min
21	120	125 μm	6 min

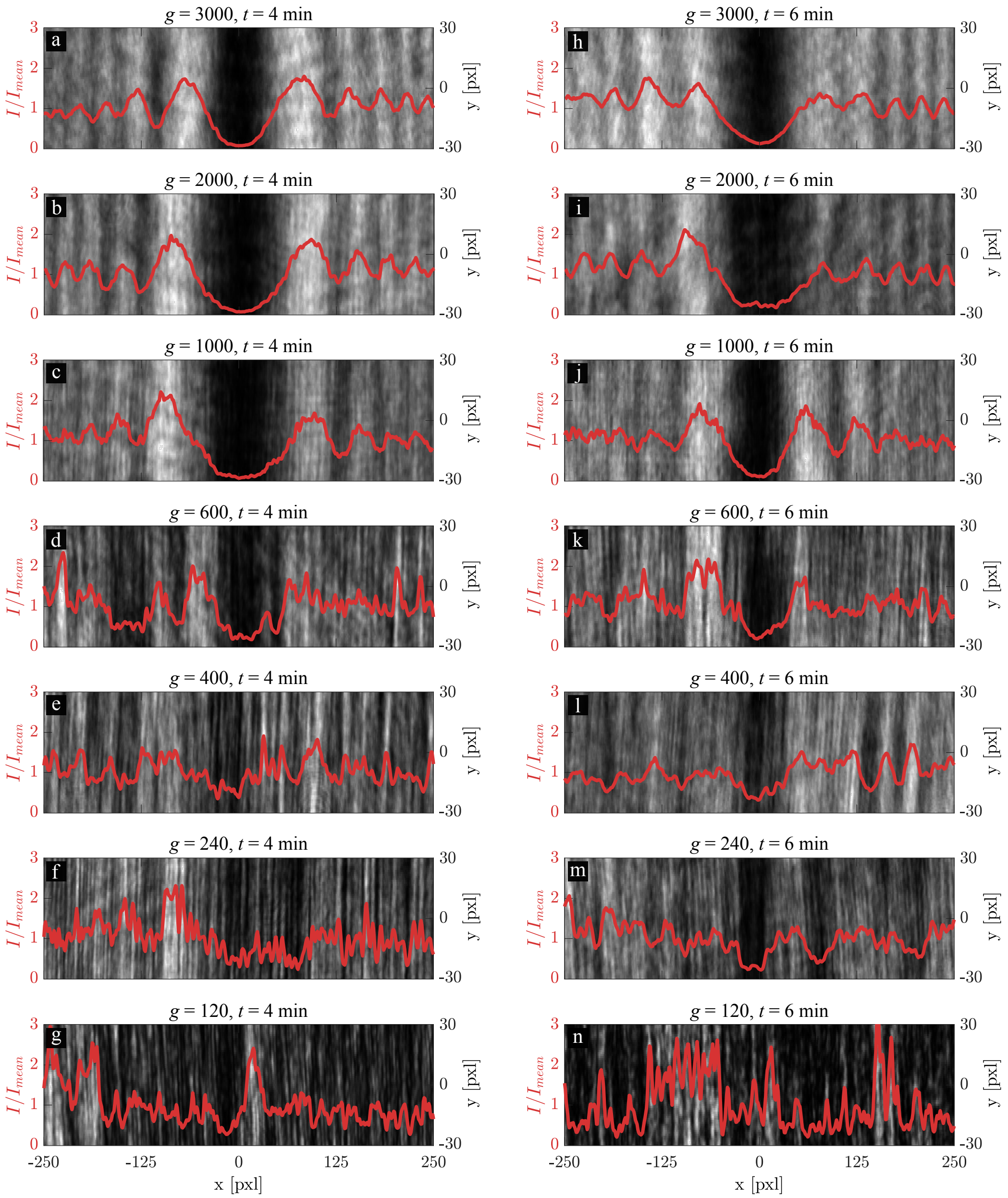

Supplementary Figure S1: Diffraction patterns and overlaid cross-sectional profiles obtained from the experiments on phase steps with different sandpapers and for (a-g) 4 min and (h-n) 6 min sanding times.
